# Achieving Universal Healthcare Coverage in a Multilingual Care Setting: Linguistic Diversity and Language Use Barriers as Social Determinants of Care in Ghana

**DOI:** 10.1177/10497323241298886

**Published:** 2024-11-28

**Authors:** Abukari Kwame

**Affiliations:** 1 College of Nursing, 248222 University of Saskatchewan, Saskatoon, SK, Canada

**Keywords:** multilingualism, linguistic diversity, universal healthcare coverage, language barriers, nurse–patient interactions, Ghana

## Abstract

The Health Sustainable Development Goal (SDG3) focuses on achieving universal healthcare coverage (UHC) through people-centered primary care and access to affordable high-quality healthcare services, medicines/vaccines, and specialized care professionals without undue financial stress. However, achieving UHC can be challenging if healthcare providers and patients cannot communicate meaningfully. Severe language barriers affect access to healthcare services. This study explores how linguistic diversity and language use barriers impact person-centered care delivery and access to healthcare services in a multilingual Ghanaian healthcare setting. Data were collected through in-depth individual interviews with patients (*n* = 17), caregivers (*n* = 11), and nurses (*n* = 11), one group interview with four patients, and participant observations. Data transcripts and field notes were inductively and manually coded and analyzed thematically. The study revealed that language barriers affect effective nurse–patient communication and interaction. Healthcare professionals and patients shop for translators and interpreters to overcome communication challenges. The study also found that healthcare professionals used medical jargon to emphasize their identity as experts despite its consequences on nurse–patient interactions and patient care. Miscommunication and misunderstanding due to language barriers derail nurse–patient therapeutic relationships and undermine patient disclosure, participation in the care process, and care quality, leading to adverse UHC outcomes. Therefore, serious attention must be paid to language use contingencies to achieve universal care, especially in resource-scared and multilingual healthcare contexts.

## Introduction

The Health Sustainable Development Goal (SDG3) aims to ensure healthy lives and promote well-being by 2030, with two of its targets focusing on achieving universal healthcare coverage (UHC) and access to safe, effective, affordable, and high-quality medicines and vaccines (United Nations General Assembly, 2015). Achieving UHC requires strong and people-centered primary healthcare systems that allow all people to access affordable and high-quality healthcare services for their health (World Health Organization [WHO], 2020).

For UHC to materialize, respect for patient rights and person-centered care (PCC) practices must be valued in healthcare delivery and clinical interactions (Miltenburg et al., 2016). The principles of PCC ensure that patients’ care needs, values, beliefs, and circumstances are respected and allowed to guide patient–provider interactions (Institute of Medicine [IOM], 2001; International Council of Nurses [ICN], 2021).

Communication and language use barriers can impede PCC when patients and care providers cannot communicate and interact meaningfully. Studies in Namibia have shown that patients experience poor care quality in multilingual healthcare settings due to language use barriers (Basimike, 2018; Mlambo, 2017). Mlambo (2017) observed that attention to multilingualism is crucial in Africa if healthcare providers must provide high-quality healthcare that meets patients’ health needs and unique individual rights and equity.

Furthermore, poor communication during patient–provider interactions can affect patients’ future access and utilization of healthcare facilities and services despite availability (Chapman, 2016; Ojwang, 2011). Poor communication, misunderstanding, and limited patient–provider interactions due to linguistic diversity can constrain PCC and UHC.

A multinational study on language needs in HIV/AIDS-related health planning and communication in healthcare policy documents in Burkina Faso, Ghana, and Senegal found minimal attention to language use in translations and limited concrete actions to address multilingualism in health communication planning (Batchelor et al., 2019). This multinational study further shows that multilingualism was not prioritized in health communication reporting around HIV/AIDS in these countries despite linguistic diversities (Batchelor et al., 2019).

Ghana is experiencing major or significant challenges in achieving the SDG3 targets and is ranked 122nd out of 166 countries (Sachs et al., 2023; Sustainable Development Report, 2022). Aside from Ghana’s low performance in the SDG3 targets, research shows that language barriers often cause disagreements and reactive violence, constraining facility-based care access, leading to a preference for home delivery (Ansah & Orfson-Offei, 2022; Dzomeku et al., 2020). Healthcare professionals in Ghana face communication barriers that negatively affect patients’ engagement in their care, leading to poor perceptions of care quality (Amoah et al., 2019; Zutah et al., 2021).

Despite significant progress in the number of healthcare facilities and healthcare personnel, the progressive healthcare financing over the years, and the creation of Ghana’s National Health Insurance Scheme to mitigate healthcare costs (Blaboe, 2019; Ofori-Agyemang, 2023), communication and language barriers continue to persist in healthcare facilities, affecting healthcare access (Ghana SDG Report, 2022). Linguistic diversity and multilingualism present challenges when accessing healthcare in Ghana.

Multilingualism, defined as the ability to have linguistic proficiency and competency in two or more languages, in linguistically diverse settings, can create avenues for linguicism (linguistic discrimination) and deprive people of their language use rights (Nordquist, 2017), especially when some languages are accorded higher status than others. Accessing healthcare in contexts where dominant and minority languages co-exist, as in the case of Ghana, can create language use discrimination and communication barriers. Evidence shows that multilingualism impacts healthcare interactions in Ghana, including patient disclosure during physician–patient clinical communication (Acquah, 2011).

The primary objective of this paper is to examine how multilingualism and communication barriers affect patient–provider clinical interactions, access to healthcare, and the achievement of UHC in Ghana. These questions are explored: (a) How do communication and language barriers affect provider–patient interactions and PCC in Ghana? (b) How can language barriers affect the achievement of UHC in the Ghanaian multilingual health context?

### Theoretical Framework

The study findings were interpreted through the lens of Positioning Theory and the Theory of Dialogue. Positioning Theory sees communication as an interpersonal activity through which people jointly construct meaning and assign rights, duties, and responsibilities to one another. Davies and Harré (1990) define positioning as the discursive production of selves when people engage in discourses. People produce and occupy positions in storylines (narratives), perform social acts through language use, position themselves and others (make moral claims), and engage in identity formation (Davies & Harré, 1990; Harré et al., 2009; McVee et al., 2018). Positioning Theory recognizes power asymmetry in interpersonal interactions as moral claims, duties, and rights (positions) are claimed or contested. This means that communication or language use becomes a discursive social practice where participants create positions and achieve positioning in storylines.

The Theory of Dialogue (Weigand, 2010, 2015, 2017) on the other hand conceives communication as a dialogue where human beings engage in a sequence of actions and reactions. Through dialogue, participants interact to come to an understanding. Weigand (2010) argues that human beings act and react to each other when negotiating meaning and understanding, which means that poor communication can create misunderstanding. The theory assumes that humans are social beings, have integrated abilities, and are goal-driven, with their needs, desires, and purposes influencing how they act in communication. Thus, communication is seen as a two-part sequence of initiative and reactive speech or action and reaction.

These theories provide analytic frames through which language use and communication barriers were explored to determine their effects on patient–provider interactions, care delivery, and UHC. Power dynamics during patient–provider communication and language use practices were examined, as the participants positioned one another and engaged in speech acts and reactions during clinical interactions. Given that humans have integrated abilities, where the act of speaking interacts with thinking, perception, and emotions (Weigand, 2010), patients and healthcare providers will respond, accept, challenge, reject, and sympathize, with each other’s positions, duties, rights, and perspectives during clinical interactions or when accessing care.

## Methodology

Data reported in this study was drawn from a larger doctoral research project that utilized exploratory qualitative research design, employing institutional ethnography (Rowland et al., 2019), interpretive phenomenology (Smith, 2017; Smith et al., 2009), and critical discourse studies (Reisigl, 2017; Reisigl & Wodak, 2016) approaches, to explore how patient–provider interaction and communication practices impact patient rights in Ghana. The broader research project, part of whose data is reported here, was aimed at understanding how the healthcare institutional culture and practices influenced nurse–patient communication and interaction, power and ideological positionings, and patient rights in clinical interactions. Participants’ experiences regarding patient–provider communication, language barriers, and patient participation in the care delivery process were critical, forming this paper’s focus. The participants’ experiences around multilingualism and language barriers to clinical interactions shed significant insights into how poor communication outcomes can adversely impact PCC and efforts to achieve UHC.

### Study Setting and Language Context

This study was conducted in a government hospital in Yendi in Ghana’s Northern Region. The community is an urban settlement linking several other districts and towns in the eastern enclave of the Northern Region. The hospital is a referral facility for Bimbila, Zabzugu-Taletale, Saboba-Cheriponi, Gushegu-Karaga, and the Mion districts as well as a clinical facility for the Health College in Yendi (DailyGuide Network, 2023). It has over 30 units and departments and a total of 433 staff serving a population of over 250,000 people across the Municipality. Primacy care, including maternal and reproductive, pediatric, emergency, surgical, and neonatal intensive care services are provided in the hospital, to promote UHC (DailyGuide Network, 2023).

The dominant native language spoken within the hospital and the Northern Region is Dagbani. However, clients who access healthcare from the hospital also speak Hausa, Fulani, Eve, Asante Twi, and Lipkakaan (the Konkomba language). The hospital serves diverse linguistic and cultural groups, reflecting the linguistic diversity in Ghana, a country with over 80 languages (Bergen, 2019; Kropp Dakubu, 2015). English, as the official language of Ghana, is used with other 11 Ghanaian languages (Dagaare, Dagbani, Ewe, Fante, Ga-Adangbe, Gonja, Gurune, Hausa, Kasem, Nzema, and Twi) across several domains, including healthcare facilities (Owu-Ewie & Eshun, 2015; Owusu-Ansah & Torto, 2013).

### Participants and Sampling

Participants for this study were nurses, patients, and caregivers (patients’ relatives who provided caring support) purposively sampled to share their experiences on the research topic. Participant recruitment was facilitated by word of mouth and through posters distributed throughout the hospital (inside patient wards and on public notice boards). Additionally, a few nurses facilitated the recruitment of patients who were fully recovered but were not yet discharged. The nurses identified these participants so the researcher could invite them to participate.

Nurses and midwives with 3 years or more working experience in the hospital were included. Caregivers and patients were included if they could engage in interviews for at least 20 minutes without compromising the patient’s health. Caregivers shared their experiences about patient–provider interactions, communication, and patient rights and did not speak on behalf of the patients they cared for. All participants were 18 years and older and provided voluntary consent to participate. Participants who expressed interest in the study were provided consent forms to read and sign. For those who could not read, the researcher explained the consent process to them in Dagbani after which they consented by thumb-printing. Detailed participants’ demographics are shown in Table 1.

**Table 1.** Participants’ Demographic Characteristics.

Characteristics	Patients	Caregivers	Nurses
Sample size	21	11	11
Marital status			
Married	9	8	9
Single	12	3	2
Age in years			
Range	18–60	19–45	26–40
Mean age	26	32	33
Sex			
Male	5	4	7
Female	16	7	4
Languages spoken by participants			
Only Dagbani	7	4	0
Dagbani + only English	3	2	1
Dagbani + English + 1 Ghanaian language	3	2	5
Dagbani + English + 2 Ghanaian languages	0	0	4
Dagbani + English + 3 Ghanaian languages	0	0	1
Dagbani + 2 Ghanaian languages	3	2	0
English + 2 Ghanaian languages	5	1	0
Ethnicity (ethnic identity)			
Dagomba	13	8	8
Eve	2	0	0
Konkomba	2	2	0
Others	4	1	3
Level of education			
No formal education	3	2	0
Basic	2	6	0
Secondary	10	1	0
Tertiary	6	2	11

*Note.* Other ethnic identities were Gonja, Fulani, Bimoba, Akan, Bono, Mamprusi, Basari, and Frafra.

### Data Collection

Data were gathered between December 2021 and April 2022 through semi-structured individual interviews (*n* = 39), one group interview (with four patients), and over 400 hours of participant observation of nurse–patient daily interactions (Saldaña, 2015) for the larger project. The researcher conducted interviews in English and Dagbani within the hospital premises in a quiet room or outside patient wards. All interviews with nurses and some patients who did not speak Dagbani (but were proficient in English) were in English. Patients and caregivers who could not speak English or did not want to be interviewed in English had their interviews in Dagbani, a language of which the researcher is a native speaker. The average interview length for patients was 21 minutes, 32 minutes for nurses, and 19 minutes for caregivers. All interviews were audio recorded and those conducted in English were transcribed verbatim. Interviews conducted in Dagbani were translated into English during transcription, an exercise the researcher has extensive experience in, as a native Dagbani speaker with full professional competency (high oral and written proficiency).

Participant observation was another data collection approach conducted during nurses’ medication rounds, during clinicians’ ward rounds, and at the nurses’ stations. Participant observations were focused on patient–provider daily interactions, nurses’ daily care routines, activities at the nurses’ station, language use practices (e.g., use of address terms, politeness marking, and facework), the spatial organization of inpatient wards, and institutional practices. The researcher undertook these observations without disrupting clinical processes/routines (Musante, 2015).

Additionally, the researcher engaged in informal chats with nurses at the nurses’ station and three formal meetings with heads of the pharmacy, laboratory, and patient records department to understand how institutional practices and processes influence care delivery. Detailed ethnographic field notes were written from the observations, informal chats with nurses, and meetings with key unit heads.

Finally, the researcher conducted one group interview lasting about 58 minutes with four patients. These participants were surgical patients who were waiting for their scheduled surgeries. They were in the same patient ward and had agreed to be interviewed together. Details about these data collection processes are reported in Kwame (2023).

Although separate interview guides were used for the different participant groups, the key interview questions and probes around communication and language barriers included (i) How would you describe your communication with nurses/patients/caregivers? (ii) What has been most challenging for you when interacting with nurses/patients/caregivers? (iii) What can cause disagreements between you and nurses/patients/caregivers? (iv) What factors make communicating or interacting with nurses, patients, or caregivers difficult? Have you experienced any difficulties talking to nurses/patients/caregivers? If so, tell me what happened, and (v) How do you think communication challenges can affect your health or the care you receive from nurses or render to patients?

### Data Analysis

Interview transcripts and field notes were coded together. Manual inductive data coding was undertaken following the strategies outlined in Elliott (2018) and Linneberg and Korsgaard (2019). To create themes, the researcher performed reflexive thematic analysis (Braun & Clarke, 2006, 2019) to identify key communication and language barriers and institutional practices from all data sets that influenced patient–provider interactions. Power dynamics and ideological positioning in nurse–patient communication and language use practices were also noted, especially regarding healthcare providers’ language behavior as the researcher read all interview transcripts and observation notes. Narratives and ethnographic descriptions were created to illustrate and organize the themes and subthemes. Five themes were developed to explain how linguistic diversity and language barriers in this multilingual healthcare context impacted patient–provider communication, care delivery, and PCC outcomes (see Table 2).

**Table 2.** A List of the Thematic Topics.

Global Theme	Supporting Themes
Multilingualism, communication, and language use barriers on patient-centered care and UHC	*No shared language*
	*Shopping for translators/interpreters*
	*Signing patient health (using sign language)*
	*Using medical jargon or everyday language (identity formation)*
	*Poor communication creates misunderstanding*

### Enhancing Rigor

Rigor, trustworthiness, credibility, and confirmability of the research findings were achieved through these credibility checks. (1) Data collection and analysis were iterative to inform other data collection processes, including participant observations and the formal meetings the researcher had with some hospital department heads. (2) Many participants (all nurses and some patients who agreed to review their transcripts after transcription) reviewed the transcripts and provided feedback (McGinley et al., 2021). The feedback mostly included clarifications on certain pieces of information and corrections of medical terminologies, which were all considered during data analysis. (3) The researcher consistently interacted with the doctoral research committee during peer debriefing, presented preliminary findings during research seminars, and shared the same preliminary findings with the hospital community to enhance the reliability and trustworthiness of the results (Forero et al., 2018). (4) The researcher used different data collection tools and analytical approaches to ensure the findings were grounded in the data (Forero et al., 2018; McGinley et al., 2021). (5) Lastly, the researcher ensured rigor through a prolonged stay in the field and immersing himself in the data collection and analysis processes (Forero et al., 2018; Musante, 2015).

### Ethical Approvals

The broader study part of which data is reported here received institutional ethical approval from the University of Saskatchewan (Beh-ID: 2690) and the Ghana Health Service Ethics Review Committee (GHS-ERC: 005/11/21). The hospital management also granted their permission, and all internal protocols were observed. Participants provided their written consent by signing or thumb-printing the consent forms, and their anonymity and confidentiality were promoted by removing all identifying information and replacing them with serial numbers and pseudonyms.

## Results

### The Study Participants

Participants of this study were nurses, patients, and caregivers. A total of 43 participants, of which 27 were females, participated in the research. Many participants were Dagomba (*n* = 29), bilinguals (*n* = 32), married (*n* = 26), and educated to some extent. Note that bilingualism and multilingualism are used interchangeably in this study to mean the ability to speak two or more languages. Also, participants’ ethnic identity describes the primary ethnic group they belonged to, thereby showing the linguistic diversity in the hospital. Table 1 provides details about participants’ demographics.

The main themes in Table 2 were developed to answer the research questions.

### No Shared Language

This theme describes the challenges of a lack of a shared common language between patients and nurses, hindering effective communication and care access, although multilingual patients, especially those who could speak Dagbani and English, faced fewer challenges interacting with nurses, as this 22-year-old female patient indicated.

I understand both Dagbani and English, so nurses who speak Dagbani to me, I understand them, also nurses who speak English to me, I understand that too.

However, many other participants experienced communication barriers due to a lack of a shared language. The following excerpts illustrated how the lack of a shared language impacted patient–provider interactions and care delivery.

I am worried that I can’t talk directly with the nurse. I will be happy to have a nurse who speaks my language so I can talk directly to him. (A 27-year-old female patient)

When you speak to me in a language that I don’t understand, then you are just wasting your time. So, when a nurse speaks to me in a language I don’t understand, I won’t even say anything to him/her. (A 26-year-old female group interview patient)

While patients became adamant or worried about the lack of a shared language to interact with nurses and other care providers, some nurses felt frustrated due to language barriers.

If you come to me as a patient, say you are a Konkomba, I understand it just a bit .… let me put it that way. So, if I am going to communicate with a Konkomba patient/caregiver, it will be very difficult. (N1)

Hmm, sometimes there is a language barrier. I don’t understand Likpakpaanl (the native language of Konkomba), but the nearby communities are Konkomba. So, the language barrier affects how we communicate or interact with them. (N10)

Not having a shared language between care providers and patients/caregivers prompted the use of translators and interpreters, whom nurses had to shop for because the hospital did not have professionally trained translators/interpreters.

### Shopping for Translators/Interpreters

Language barriers affected many nurse–patient–caregiver interactions. To manage the problem of no shared language, nurses, patients, caregivers, and clinicians shopped for translators and interpreters to enhance communication. Different individuals including nurses, caregivers, cleaners, visitors, patients, and the researcher were often contacted to translate/interpret for nurses or patients, as noted here.

When a patient comes and doesn’t understand your language, we try to find a nurse who understands the patient’s language. Sometimes, we even go to other wards to find a nurse or anybody who understands the language, and we will use that person to translate for us. But it’s not always effective because the meaning may change. It’s not also direct interaction with the patient. (N3)

A patient came to the labour ward with her mother when I was observing nurse-patient interactions in the ward. They both didn’t understand English, and the nurses on duty could not speak Dagbani. So, I (the researcher) was invited to translate/interpret for the patient. (Field note documented, February 23, 2022)

Having to rely on translators or interpreters to interact with nurses and other care providers helped many patients, but for others, the interaction was muted when there was no person to interpret or translate, as this 23-year-old male patient narrated: “Today, some nurses came to me. They were trying to interact with me in Dagbani, but I told them I don’t understand Dagbani.” The following excerpts further illustrate patient and caregiver perspectives on using translators and interpreters to navigate language barriers in the hospital.

The nurses will try to find someone who understands the patient’s language. For instance, if a patient is Konkomba by tribe, they will find someone who understands the Konkomba language to do [the] translation, and if it’s Dagbani or English, they’ll get someone to translate for the patient or the nurse. (A 22-year-old female patient)

One of the nurses here doesn’t understand Dagbani. So, when s/he is going to talk to you, the nurses will call one of their colleagues to come and translate for her/him. (A 22-year-old female patient)

Despite the importance of engaging with these translators/interpreters, using non-medically trained translators and interpreters could impact the accuracy of health data and patient disclosure and privacy as third persons unrelated to the patient join the interactions.

### Signing Patient Health

When nurses could not find anybody to translate/interpret for them or feared that patient privacy might be affected, they resorted to using sign language and/or gestures. The narrative below reveals how some nurses manage their interaction with patients when they could not find an interpreter or translator to mediate the interaction.

There was a day a patient was brought here; he was a Fulani. I couldn’t speak the language, nor could the patient speak my language. Looking around, there was only one young man who could help. I spoke Dagbani to him, but seeing that the case was sensitive, and bringing in someone to interpret, I wasn’t sure, whether they would talk about the patient’s condition later. So, in that instance, I had a big challenge. So, I resorted to using sign language with the patient, and with the sign language too, I wasn’t sure if I was recording the right information from the patient. It was one of the days I had a serious challenge communicating with a patient. (N4)

In addition, the following field note demonstrates how language barriers led to care providers using sign language and gestures to communicate with patients and caregivers.

Today, the language barrier issue came up while I was in the patient ward. The nurses shared their experiences interacting with patients and caregivers who don’t understand English. They said students and regular nurses translate for patients, especially Fulani and Zambarima clients. The nurses indicated that sometimes, they use non-conventional sign language to interact with patients or their caregivers. One nurse said, “Sometimes I have to demonstrate by touching the naval area if I want to tell the caregiver something relating to the umbilical cord.” (Field notes documented on January 23, 2022)

Another instance of using sign language was observed in the outpatient department (OPD), as noted herein.

On Friday, a Fulani woman, his son, and a young girl came to the OPD. The girl was the patient, but the woman and her adult son who accompanied the patient could not speak Dagbani, and no one at the nurse station spoke Fulani to translate/interpret for them. The nurses and the woman managed to interact using signs and gestures. In the end, the nurse directed them to the lab. (Field note, documented March 25, 2022)

The above data describe how language barriers affected patient–provider communication and interactions, sometimes forcing healthcare professionals to use unconventional sign language.

### Using Medical Jargon or Everyday Language

Although many nurses claimed that they use everyday language when interacting with their clients, effective communication was often impeded when nurses failed to speak to patients and caregivers in simple language. The nurses’ use of medical language was influenced by two crucial factors: to appear serious regarding patients’ conditions and/or for identity formation. A nurse shared her perspective on using medical jargon or simple language when communicating with patients and caregivers.

I use everyday language to interact with patients. If you use medical jargon, they won’t understand, [ok]. If you base your talk on the professional aspect, you will use your professional terms, which they won’t understand. (N5)

While simple everyday language aided understanding, other nurses used medical jargon to appear serious or demonstrate that they have the expertise to care for patients.

When patients come like that, they are anxious. So, if you don’t add the professional and only use everyday language with them. … if the condition is not critical, your language might not offend the patient, but, if the patient comes in a critical condition, you must add professional language to it, because if you speak the everyday language to the person, they might not see the severity of the case. (N6)

Maybe you need the patient to understand something, so you come in a professional way to make him understand. If you just come and say anyhow, (laughs) they will not get you. You don’t come with a uniform identity. You must know what you are talking about so that your patient will believe you first and understand that you are really a nurse, with some knowledge to say what you are saying to him or her. If not, she will not buy that idea. (N3)

Using medical jargon affected patient–provider interactions, potentially impacting patients’ understanding of their health conditions.

### Poor Communication Creates Misunderstanding

Language barriers affect therapeutic interactions and could lead to conflicts and misunderstandings between patients/caregivers and nurses. For instance, a nurse said the following about the value of effective communication.

The way or manner a nurse speaks to patients, without even giving them any treatment, can tell the patient that the nurse is caring. For some patients, it’s not just the medicine they receive, but sometimes they need psychological support [in the form], such as someone speaking to them in a good manner. (N11)

Despite the above value of communication in healthcare delivery, participants reported that poor communication often caused misunderstandings and conflicts between nurses and patients/caregivers, as the following excerpts illustrate.

Patients and caregivers come from different societies. So, for some patients/caregivers, how nurses talk to them may not be appropriate. So, when nurses talk inappropriately to patients or caregivers, it can make them angry and cause disagreements or fights. (A 28-year-old female caregiver)

Our tongue is a weapon, yet people don’t train their tongues well. If people are careful, they won’t speak bad or hurtful words to others. Even if you do something that will hurt another person, but you use “please,” the person might not even feel the pain much. (A 24-year-old female patient)

The power of communication is expressed metaphorically as a weapon when the tongue is not used correctly. Aside from poor communication affecting therapeutic interactions and relationships, the data revealed that it could also cause dissatisfaction among patients and caregivers, as a nurse noted.

Yeah, sometimes (clears throat), for the community here, I know, it’s because of poor communication that patients might not be satisfied. But they are fine once you open yourself and communicate well with them. (N7)

Other nurses lamented the impact of language barriers on patients’ understanding of their healthcare conditions, as noted below.

When we say hypertension, in Dagbani, bԑ yen yelmi ni ʒiduli. Bԑ ni yԑli ni ʒiduli maa (translated: in Dagbani, they will say the blood has risen). Patients may understand it to mean the quantity of blood is too much (we both laugh). So, we try to make them understand that it’s actually about the blood pressure, not the [quantity]. (N1)

The above nurse believes that sharing a common language with patients is not enough because medical concepts and terminologies cannot be easily translated into native Ghanaian languages, underscoring how communication and language use barriers affect PCC and patient–provider interactions.

## Discussion

This study explored multilingualism and language barriers in patient–provider interactions and how that impact PCC and efforts to achieve UHC in Ghana. The study found that healthcare professionals and patients often lack a shared language for effective interaction. To manage this challenge, untrained translators and interpreters were shopped for while at other times sign languages were used to manage communication challenges. Medical jargon and communication barriers created misunderstandings between patients and healthcare professionals, positioning patients peripherally in the care process.

This study found that the lack of a shared language between healthcare providers and patients was a significant barrier to therapeutic interactions and care outcomes as reported in previous studies (Johnsson et al., 2018; Ostaszkiewicz et al., 2020). Moreover, poor patient–provider communication due to language barriers increases the risk of misdiagnosis (Deumert, 2010). Since communication is dialogic, where people act and react to others, communication barriers can negatively affect information quality in provider–patient interactions. In Ghana, deaf patients often make repeated visits to healthcare facilities because of communication barriers, predisposing them to wrong medications and delayed care (Appiah et al., 2018).

Although interpreters/translators facilitate patient–provider interactions when patients and providers lack a common language to interact. However, medical interpretation presents challenges too, such as meaning change, privacy concerns, and mediated interactions (Goldhirsch et al., 2021; Krupic et al., 2016). Using untrained interpreters and translators is tasking and leads to complicated clinical interactions as found in this current study and previous studies (Basimike, 2018). For instance, it is reported that medical doctors in Namibia who spoke only English relied on nurses, friends, and other patients for medical interpretations (Basimike, 2018). However, these untrained interpreters lacked contextual knowledge, which affected doctors’ judgment (Basimike, 2018). This situation is further compounded when healthcare providers must explain medical terminologies in local languages or describe native illnesses in English using the biomedical language, as found in this current study and the literature (Karidakis, 2021).

Patient engagement in their care process is a crucial PCC principle, which communication and language barriers can affect. This study observed that communication and language barriers impede patient/caregiver participation in the care process, resulting in poor patient engagement in the care process (Al Shamsi et al., 2020). Patients indicated that if a nurse spoke to them in a language they did not understand, the nurse would be wasting their time. This situation could generate negative perceptions of care when nurses cannot provide efficient information to support patients’ self-care management (Appiah et al., 2018; Kreps, 2018; Kreps et al., 2020). Besides, language barriers hamper the dialogic nature of patient–provider interactions. Moreover, communication difficulties exacerbate care disparity, patient neglect, discrimination, and delays in receiving PCC among patients (Appiah et al., 2018). The Dialogue Theory holds that communication is a joint initiative of action and reaction where people exchange information during social interaction to meet each other’s needs (Weigand, 2010, 2015). According to this theory, speaking, thinking, emotions, and cognitions are integrated human abilities which are activated during communication; as a result, participants who do not share the same language (face a language barrier) (Han et al., 2020) can experience miscommunication and emotional disequilibrium leading to reactive violence.

Also, this study discovered that medical jargon constitutes identity formation among many healthcare professionals, as reported in the literature (see Ampofo et al., 2022). However, this language behavior affects healthcare access and quality. Healthcare providers occupy power and privileged positions because of their educational and professional expertise; hence, they must reflect on their use of medicalized language (medical jargon) when communicating with patients to encourage patient participation in the care process. In line with this reasoning, the ICN (2021, p. 2) requires healthcare professionals to provide “understandable, accurate, sufficient and timely information in a manner appropriate to the patient’s culture, linguistic, cognitive, and physical” care and health needs.

Furthermore, the study found that multilingualism and communication barriers constrain nurse–patient therapeutic relationships, forcing healthcare providers to use informal sign language. However, Yakong et al. (2010) argued that using non-standardized signs creates misunderstanding and mistrust in patient–provider interactions. Previous studies have also shown that providers and patients may withhold essential information from each other due to language and communication barriers or improper signing (Adade et al., 2023; Yakong et al., 2010). Based on Dialogue Theory, when perception and emotions intersect with language use and communication barriers, patient–provider conflicts become imminent (Weigand, 2015).

Moreover, linguistic marginalization is confirmed as a significant barrier to accessing health services and information in the literature (Showstack et al., 2019), supporting the findings of this current study. In addition, research in Guatemala has shown that language barriers promote health inequities, with physicians and nurses denying patients quality care because they do not speak the majority language (Cerón et al., 2016). Cerón et al. (2016) reported that poor proficiency in English among these patients had positioned them to receive substandard care, showcasing multilingualism as a social determinant of care.

### Health Implications and the Way Forward?

Healthcare professionals must be equipped with various communication strategies to engage well with their clients. Using sign language, lip reading, assistance from a family member or friend, and short writing facilitates communication with deaf and hearing-impaired patients (Appiah et al., 2018). Although a sign language course has been introduced into the nursing curriculum in Ghana (Adade et al., 2023), healthcare professionals need other communication strategies and basic interpretive and translation skills to interact with patients who do not share a common language. Effective communication enhances trust and transparency between healthcare professionals and care consumers which can be achieved when healthcare professionals possess excellent communication competencies. Effective communication can build strong dialogic and therapeutic relations in clinical interactions. Therefore, healthcare education and training in Ghana must emphasize medical interpretation and translation and how culture and multilingual contexts affect communication.

Linguistic diversity should be seen as a social determiner of care, especially in contexts where such diversities affect meaningful interpersonal interaction and communication (Edu-Buandoh, 2016). Cultural and language differences in Ghana affect provider–patient interactions, especially where healthcare professionals lack minimal language proficiency in a local language to interact with patients and caregivers who cannot speak English. This situation adversely affects healthcare access and the quality of care, thereby minimizing the realization of the UHC mandate in Ghana.

A rethink in healthcare language policy and when posting healthcare professionals is crucial in Ghana to reduce the language use challenges nurses and patients face. Although code-switching, code-mixing, and using sign/body language, gestures, and interpreters can help resolve some language use challenges reported in this study, other problems persist. I agree with other scholars that nurses and other healthcare professionals must have minimal language proficiency in the local language of the place they are posted to (Abdulai et al., 2019) or be interested in learning the local language. Posting nurses to serve at places where they know the local language may come with some difficulties due to the limited nursing workforce in Ghana. However, it has long-term benefits.

Lastly, a national medical survey should be undertaken to document names of local illnesses, their descriptions and medical conditions, and community case definitions to compile a Ghanaian-based multilingual medical dictionary. This dictionary can equip healthcare providers with knowledge of local illnesses. For example, Nyaaba et al. (2020) observed that language barriers are a significant obstacle to hypertension control in Northern Ghana because translating hypertension to people in the local languages (Dagbani or Gurune) is challenging thereby limiting patients’ knowledge of the illness.

## Conclusion

This study explores the impact of multilingualism and language barriers on PCC, care delivery, and UHC in Ghana and found that language barriers adversely affect patient–provider interactions and patient care access. Multilingualism in Ghana due to linguistic diversity creates communication barriers resulting in poor healthcare access and negatively impacting Ghana’s UHC priorities and PCC outcomes. Lack of shared language between patients and nurses, using medical jargon, untrained medical interpreters and translators, and informal sign language all affect effective patient–provider communication. I conclude that multilingualism is a social determiner of care in Ghana; hence, communication and language use planning must be prioritized in healthcare policy, education, training, and practice.
